# Right Dose Right Now: bedside data-driven personalized antibiotic dosing in severe sepsis and septic shock — rationale and design of a multicenter randomized controlled superiority trial

**DOI:** 10.1186/s13063-019-3911-5

**Published:** 2019-12-18

**Authors:** Luca F. Roggeveen, Lucas M. Fleuren, Tingjie Guo, Patrick Thoral, Harm Jan de Grooth, Eleonora L. Swart, Thomas L. T. Klausch, Peter H. J. van der Voort, Armand R. J. Girbes, Rob J. Bosman, Paul W. G. Elbers

**Affiliations:** 1 Department of Intensive Care Medicine, Amsterdam Medical Data Science (AMDS), Research VUmc Intensive Care (REVIVE), Amsterdam Cardiovascular Science (ACS), Amsterdam Infection and Immunity Institute (AI&II), Amsterdam UMC, Location VUmc, Vrije Universiteit Amsterdam, De Boelelaan 1117, 1081 HV Amsterdam, The Netherlands; 2 Department of Clinical Pharmacology and Pharmacy, Amsterdam UMC, Location VUmc, Vrije Universiteit Amsterdam, De Boelelaan 1117, 1081 HV Amsterdam, The Netherlands; 3 Department of Epidemiology and Biostatistics, Amsterdam UMC, Location VUmc, Vrije Universiteit Amsterdam, De Boelelaan 1117, 1081 HV Amsterdam, The Netherlands; 4 Intensive Care Unit, OLVG Oost, Oosterpark 9, 1091 AC Amsterdam, The Netherlands

**Keywords:** Personalized medicine, Antibiotic dosing, Clinical decision support, Intensive care medicine, Pharmacokinetics, Data science, Sepsis

## Abstract

**Background:**

Antibiotic exposure is often inadequate in critically ill patients with severe sepsis or septic shock and this is associated with worse outcomes. Despite markedly altered and rapidly changing pharmacokinetics in these patients, guidelines and clinicians continue to rely on standard dosing schemes. To address this challenge, we developed AutoKinetics, a clinical decision support system for antibiotic dosing. By feeding large amounts of electronic health record patient data into pharmacokinetic models, patient-specific predicted future plasma concentrations are displayed graphically. In addition, a tailored dosing advice is provided at the bedside in real time. To evaluate the effect of AutoKinetics on pharmacometric and clinical endpoints, we are conducting the Right Dose Right Now multicenter, randomized controlled, two-arm, parallel-group, non-blinded, superiority trial.

**Methods:**

All adult intensive care patients with a suspected or proven infection and having either lactatemia or receiving vasopressor support are eligible for inclusion. Randomization to the AutoKinetics or control group is initiated at the bedside when prescribing at least one of four commonly administered antibiotics: ceftriaxone, ciprofloxacin, meropenem and vancomycin. Dosing advice is available for patients in the AutoKinetics group, whereas patients in the control group receive standard dosing.

The primary outcome of the study is pharmacometric target attainment during the first 24 h. Power analysis revealed the need for inclusion of 42 patients per group per antibiotic. Thus, a total of 336 patients will be included, 168 in each group. Secondary pharmacometric endpoints include time to target attainment and fraction of target attainment during an entire antibiotic course. Secondary clinical endpoints include mortality, clinical cure and days free from organ support. Several other exploratory and subgroup analyses are planned.

**Discussion:**

This is the first randomized controlled trial to assess the effectiveness and safety of bedside data-driven automated antibiotic dosing advice. This is important as adequate antibiotic exposure may be crucial to treat severe sepsis and septic shock. In addition, the trial could prove to be a significant contribution to clinical pharmacometrics and serve as a stepping stone for the use of big data and artificial intelligence in the field.

**Trial registration:**

Netherlands Trial Register (NTR), NL6501/NTR6689. Registered on 25 August 2017.

European Clinical Trials Database (EudraCT), 2017-002478-37. Registered on 6 November 2017.

## Background

Antibiotics are a cornerstone in the treatment of sepsis and septic shock [1]. Their early and appropriate use has been associated with improved survival [2, 3]. However, mortality rates remain as high as 40% for patients with severe sepsis or septic shock [4, 5], despite major efforts to improve sepsis treatment [6, 7]. This is particularly alarming as the incidence of sepsis continues to increase [8].

Inadequate antibiotic dosing may be an important modifiable cause for the morbidity and mortality of sepsis. Indeed, there is a strong rationale for optimization of antibiotic exposure in septic patients given the robust relationship between antibiotic exposure and bacterial killing [9]. However, achieving and maintaining adequate antibiotic exposure in critically ill septic patients is challenging as their pharmacokinetic profiles are often markedly changed. For example, their clearance may be increased by their hyperdynamic circulation; their volume of distribution may be affected by the large quantities of fluid resuscitation they may receive; and their prevalent organ dysfunction and ensuing organ support are known to modify both [9]. It is of particular importance that these changes vary greatly between patients as well as in the same patient over time. The Defining Antibiotic Levels in Intensive care (DALI) study [10–12] has confirmed the severity of this dosing challenge in this population. Up to a 500-fold variation in antibiotic concentrations in critically ill patients was observed. Also, fewer than half of patients achieved the optimal pharmacodynamic target which was associated with a decreased likelihood of attaining clinical cure.

Despite these considerations, intensive care physicians continue to rely on standard dosing schemes for prescribing antibiotics. This may be caused by the fact that clinically relevant pharmacokinetic knowledge on antibiotic dosing among intensive care professionals is insufficient [13]. For example, although antibiotic doses are often routinely reduced in the case of impaired renal function to avoid toxicity, doses are rarely increased for the well-known risk factors for underdosing such as young age, large body weight, renal hyperfiltration and septic shock [9]. Depending on patient characteristics, the clinical course and supporting therapies, routine dosing may therefore either result in underdosing and/or drug-related toxicity during the course of intensive care treatment. Toxicity may lead to excess morbidity [14], while underdosing may result in increased antimicrobial resistance through selection pressure [15], and, more importantly, in suboptimal treatment and excess mortality.

Existing solutions that address these challenges include therapeutic drug monitoring (TDM) and dosing nomograms. However, both have significant drawbacks [16]. Nomograms fail to acknowledge the complexity of altered and changing pharmacometric profiles in critically ill patients. TDM relies on manual data entry, plasma-level determination and interpretation by pharmacists. As such, TDM dosing advice is not available directly and immediately at the bedside. Paradoxically, TDM dosing guidance only becomes available after the administration of several doses, while adequate exposure may be needed right from the start of therapy. This may explain why TDM studies on relevant outcomes including mortality and cost-effectiveness are scarce and have produced mixed results [17, 18].

To address these challenges, we now propose an innovative solution that extracts electronic health record data in real time to personalize antibiotic dosing. This is useful as these electronic health records have now become rich data warehouses, especially in the context of intensive care medicine. Large amounts of precise patient information that determines their pharmacometric profile are constantly and instantly available. Therefore, we set out to develop AutoKinetics software that connects with every modern electronic health record. AutoKinetics uses all available data at the time of prediction to forecast future drug plasma levels using pharmacometric models. These typically represent individual patient characteristics including parameters from connected monitors, life support devices as well as laboratory values.

AutoKinetics is reminiscent of TDM, albeit without its drawbacks and with two distinct advantages. First, the direct and automated connection between AutoKinetics and the electronic health records circumvents manual data entry, which reduces error rates and may improve the accuracy of plasma concentration predictions. Second, through its integration with the electronic patient record, dosing guidance is always available immediately and enables prompt response to the patient’s clinical course. As such, AutoKinetics may be regarded as an expert system and a stepping stone for the use of artificial intelligence with big data in the domain of clinical pharmacometrics.

**Fig. 1:**
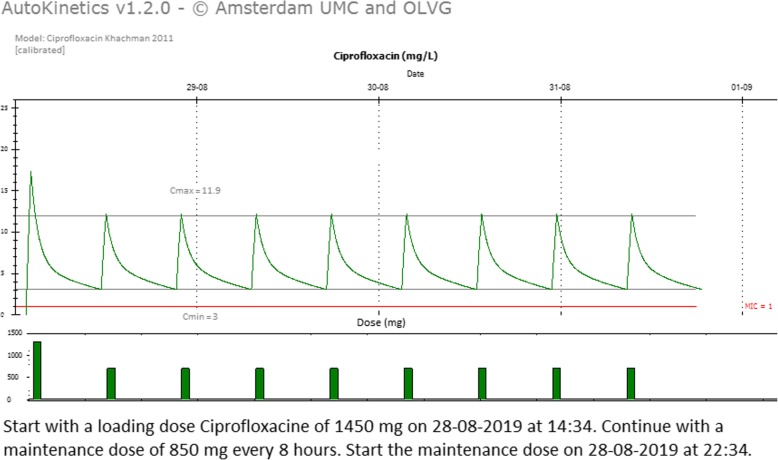
Screenshot from AutoKinetics presenting real-time predictions and dosing advice. C_max_ maximum concentration, C_min_ minimum concentration, MIC minimal inhibitory concentration

As illustrated in Fig. 1, the AutoKinetics user interface provides a graphical display of predicted past and future plasma concentrations. In addition, it also provides written and graphical dosing advice. Finally, the software automatically uses Bayesian maximum a posteriori estimation to adapt the predictions based on plasma drug samples, if available. As further detailed in the following, all dosing recommendations are based on the most appropriate pharmacometric targets, which themselves depend on the antibiotic class and known or presumed bacterial susceptibility.

It is our objective to evaluate the effect of providing bedside data science-driven decision support by AutoKinetics to healthcare professionals treating critically ill septic patients. To evaluate our hypothesis that bedside dosing guidance by AutoKinetics will improve antibiotic plasma-level target attainment and patient-relevant outcome measures, we designed the Right Dose Right Now study, a randomized controlled superiority trial.

## Methods/design

The Right Dose, Right Now trial is a multicenter, randomized controlled, two-arm, parallel-group, non-blinded, superiority trial. It is designed to investigate the effect of providing intensive care professionals with bedside, data-driven, individualized decision support from our AutoKinetics software on antibiotic plasma-level target attainment and patient-relevant clinical outcome measures.

**Fig. 2:**
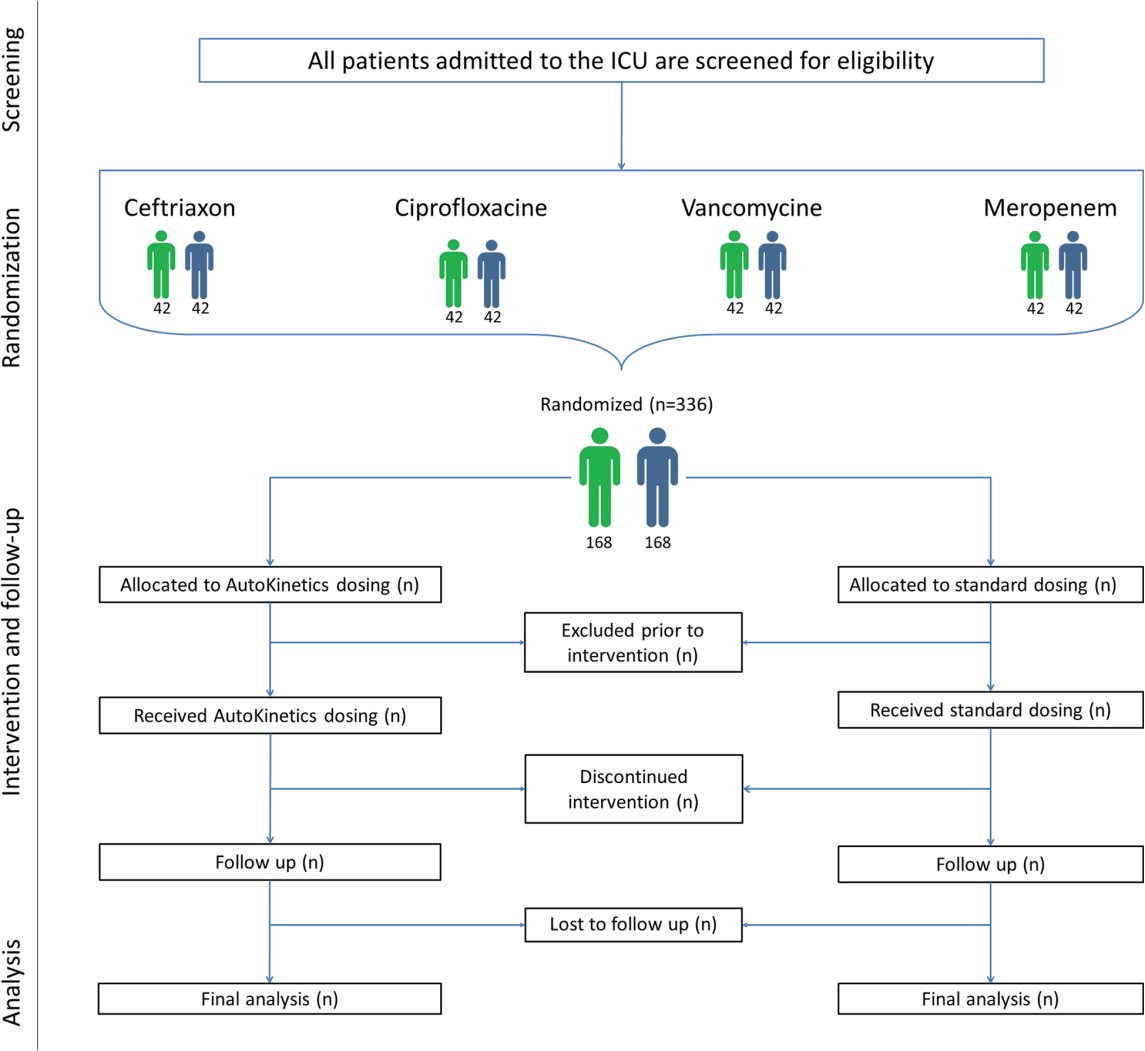
Flow diagram for the trial. ICU intensive care unit

The trial is being conducted at the Department of Intensive Care Medicine of each of two hospitals in Amsterdam, The Netherlands: Amsterdam UMC, location VUmc, a university medical center with 22 intensive care beds; and OLVG, location Oost, a large teaching hospital with 18 intensive care beds. Both are tertiary referral centers for intensive care medicine. Figure 2 shows the flow diagram of the trial.

### Eligibility criteria

This study will recruit critically ill septic patients who require antibiotic treatment. All patients meeting the following criteria will be eligible for inclusion:

- Age 18 years or older
- Intensive care treatment
- Suspected or confirmed infection
- Clinical decision to start antibiotic therapy
- One or both of the following indices of disease severity:
  - ○ Suspected or confirmed serum lactate greater than 2 mmol/L
  - ○ Requirement for any vasopressor support in any dose

### Study antibiotics, pharmacokinetic models and dosing targets

In this study we focus on four of the most frequently prescribed antibiotics for treating sepsis in the participating centers. These are the β-lactam antibiotics ceftriaxone and meropenem; the fluoroquinolone ciprofloxacin; and the glycopeptide vancomycin. Inadequate antibiotic exposure occurs with a frequency of up to 60% for all of these antibiotics [10, 12] and their appropriate dosing has been suggested to improve outcome [9].

An extensive literature review was conducted to find the most suitable pharmacometric models published for the study of antibiotics in intensive care. Candidate models were tested using retrospective and prospective antibiotic plasma-level data. A further prospective analysis was done to evaluate these models in our patient population using data from routine sampling. Finally, by means of a model validation study for each antibiotic, the most adequate model was chosen and parameters were calibrated prior to implementation. The results of model evaluations and calibrations will be reported separately.

Dosing targets are based on results from clinical and preclinical studies, and focus on the prevention of underdosing as this may lead to excess mortality. For the β-lactam study antibiotics, the time that the concentration or free concentration remains above the minimum inhibitory concentration of the bacteria involved (fT > MIC) was selected. For vancomycin and ciprofloxacin, this index is the area under the time concentration curve divided by the minimum inhibitory concentration of the bacteria involved (fAUC / MIC) [9]. Dosing recommendations are generated using the selected models and can take into account measured or presumed minimum inhibitory concentrations. Dosing targets are based on results from clinical and preclinical studies, and focus on the prevention of underdosing as this may lead to excess mortality. Table 1 presents an overview of the chosen models, dosing targets and current routine dosing practice for each of the study antibiotics.

**Table 1:** Antibiotic pharmacometric targets

Antibiotic	Pharmacokinetic model	AutoKinetics dosing target	Routine practice at Amsterdam UMC, location VUmc	Routine practice at OLVG Oost
Ceftriaxone	Garot et al., 2011 [19]	100%T > 4 × MIC	2000mg every 24 h	2000mg every 24 h
Meropenem	Muro et al., 2011 [20]	100%T > 4 × MIC	1000mg every 8 h	1000mg every 8 h
Ciprofloxacin	Khachman et al., 2011 [21]	AUC0–24/MIC > 125	400mg every 8 h	400mg every 12 h
Vancomycin	Roberts al., 2011 [22]	AUC0–24/MIC > 400	1000mg every 24 h + TDM	1000mg every 24 h + TDM

Pharmacometric models, dosing targets and routine dosing for the study antibiotics. All model parameters were calibrated prior to implementation. For meropenem and ciprofloxacin, routine dosing includes a dose reduction by 50% and an increased dosing interval to 2dd if the estimated glomerular filtration rate is less than 30 ml/min/1.73 m^2^. For vancomycin, routine dosing includes dose adaptation using therapeutic drug monitoring after 1–3 days at Amsterdam UMC, location VUmc and after every 24 h at OLVG Oost. Vancomycin is administered by continuous infusion at OLVG Oost

*AUC* area under the time–concentration curve, *MIC* minimal inhibitory concentration, *TDM* therapeutic drug monitoring, *100%T* hundred percent of time

### Recruitment

Screening and inclusion will be performed by the attending physicians. For this, our AutoKinetics software features an inclusion and randomization module instantly available at the bedside at the click of a button. This is essential to ensure that the treatment team is able to follow dosing advice right from the start of antibiotic therapy.

Patients are primarily included for one of the four study antibiotics, even if they are treated with more of them. If multiple eligible antibiotics are started at the same time, the choice of antibiotic for primary inclusion is left at the discretion of the physician. For example, if treatment with both ceftriaxone and ciprofloxacin is indicated, the patient may be included for either one of them.

### Randomization

After inclusion, the patient will be allotted to the control group or intervention (AutoKinetics) group. In the AutoKinetics group, the treating healthcare professionals will receive personalized antibiotic dosing advice. Importantly, regardless of the antibiotic the patient is included for, the treatment team will receive advice for all study antibiotics. In the control group, no such advice is provided and the treating healthcare professionals are advised to prescribe standard dosing. The electronic randomization module, which is incorporated into the AutoKinetics software, uses a 1:1 allocation ratio with stratification by study center, gender, and age with a cutoff point of 65 years using minimization techniques [23].

### Blinding

For feasibility reasons, the treatment team will not be blinded to the study intervention. Patients are also not blinded to the intervention, although it is unlikely that these critically ill and frequently unconscious patients will be aware of which dose is administered. Plasma-level determinations that will be used to assess the study effect will not be available to the treatment team. For vancomycin, routine therapeutic drug monitoring is used at both study sites. Those plasma levels are determined using a separate workflow in addition to the study samples and will be available in both groups as per standard practice. To control for ascertainment bias, the outcome assessors and data analysts will be blinded.

### Participant timeline

Patients treated in the ICU are included as soon as they meet the inclusion criteria. They stay included for the duration of their hospital admission. If a patient is readmitted to the ICU, they stay within their allocated treatment arm. In the rare event that patients who are first included in the ICU of one participating hospital are then readmitted to the ICU of the other participating hospital as part of a new hospital admission, these patients may theoretically be included in the study again and allocated to a possibly different treatment arm again if they meet the inclusion criteria.

For patients who are included in the AutoKinetics study arm, it is at the physician’s discretion to follow the advice. They have to manually enter the dose order into the electronic medical record. Physicians are recommended to check AutoKinetics at least once per shift, so at least three times per day. Physicians are asked to provide feedback through the user interface of AutoKinetics, especially if they choose not to follow the advice. AutoKinetics provides advice on dose and dose interval; the duration of antibiotic treatment and choice of antibiotics are left to the physician. In both groups, all other treatments are also left to the treatment team.

For all patients, blood sampling will be timed right after dosing, halfway through the dosing interval and right before the next dose for the first dosing interval; and daily before a next dose for the following dosing intervals. In the case of continuous infusions, sampling will be performed 1 h after a loading dose and at least daily thereafter. At one of the study sites (Amsterdam UMC, location VUmc), antibiotic plasma concentrations are also monitored as part of a continuous quality control project. Their results are not available to the treating physicians. We will use these results in addition to the samples specifically taken in the context of the present study.

For patients in the AutoKinetics study arm, antibiotic dosing is changed back to dosing according to clinical guidelines at ICU discharge. Dosing according to AutoKinetics is reinstated in the case of readmission to the ICU within the same hospital admission.

At hospital discharge, and at 6 months after hospital admission, surveys assessing quality of life and societal costs will be carried out as further specified in the following. Therefore, the maximum window in which a patient is included in the study is the duration of hospital admission plus 6 months.

### Sample size

In the DALI study [10], up to 60% of patients did not reach the PD target. We expect that it is possible and clinically relevant to reduce this percentage to 30% based on results from an active TDM study, which showed a reduction in dose changes from 80 to 49% [24] and preliminary data from our early clinically used version of AutoKinetics [16]. Power analysis (α = 0.05, 1 – β = 0.80) shows a required sample size of 42 patients per group, per antibiotic, based on a reduction from 60 to 30% failure to attain pharmacometric targets. Inclusion will be stopped when the groups are full or when funding ends. If patients have been randomized to the AutoKinetics group, physicians will continue to receive dosing advice for all study antibiotics, including those that the patient was not primarily included for, even if the required number of inclusions has already been reached for those antibiotics.

### Consent

To avoid any treatment delay, the ethics committee has approved initial inclusion under deferred consent as critically ill patients are often incapacitated. However, informed consent will be sought from the patient’s legal representative by the research team within 48 h following inclusion. The consent form explicitly describes that the study may have already commenced under deferred consent and that the patient will be withdrawn from the study should consent not be given. In select cases, patients may be able to provide informed consent themselves. If not, additional informed consent will be sought from the patient after discharge from the intensive care department. To avoid selection bias, all patients who die within 48 h following inclusion and before informed consent has been obtained remain in the study. All research team members have obtained Good Clinical Practice certification or its equivalent.

### Primary endpoint

The primary endpoint is pharmacometric target attainment during the first 24 h following randomization for the antibiotic that patients are randomized for. This target has been defined as 75%-T0–24 > 4 × MIC for ceftriaxone and meropenem, where 75%T0–24 denotes 75% of the time (thus, this is 75% of the target used for dosing recommendations); and as AUC0–24/MIC > 300 for vancomycin or AUC0–24/MIC > 94 for ciprofloxacin, which is also 75% of the target used for dosing recommendations. The area under the time–concentration curve is calculated by the trapezoidal rule for 1-min time intervals. We assume a concentration of 1 mg/ml for the MIC based on the consistent pattern of low antimicrobial resistance in the Netherlands unless the MIC has been specifically determined and electronically transferred to AutoKinetics.

### Secondary pharmacometric endpoints

AutoKinetics is designed to reach desired antibiotic plasma levels faster as well as to better maintain desired antibiotic plasma levels during the course of antibiotic therapy. We therefore define our two pharmacometric secondary endpoints as the fraction of plasma-level target attainment during an antibiotic therapy course (%-TA) and the time to attainment of plasma target levels (time to TA).

The %-TA is the fraction of days of the antibiotic therapy course for which the patient achieves the pharmacometric targets as defined in the primary endpoint. The start of antibiotic therapy is defined as the time of randomization. The end of therapy is defined as either: the moment of the last dose plus the dosing interval; the moment the physician stops the antibiotic order; the moment the antibiotic order expires without restarting within 48 h; or the end of ICU admission.

For ceftriaxone and meropenem, the time to TA is defined as the time until a concentration of 4 × MIC or greater has been reached for 75% of a 24-h time period For vancomycin and ciprofloxacin, the time to TA is defined as the time until 75% of the targeted AUC/MIC has been reached during 24 h. As the targeted AUC0–24/MIC is 400 h for vancomycin and 125 h for ciprofloxacin, 75% of those targets amount to 300 h and 94 h respectively.

### Exploratory pharmacometric endpoints

We will report actual pharmacometric targets that are reached in the first 24 h and over the course of antibiotic therapy, either as a percentage of time above MIC for ceftriaxone and meropenem or as the AUC / MIC for ciprofloxacin and vancomycin. In addition, we will explore target attainment using different predefined percentages. These predefined percentages are 25%, 50% and 100%. For the β-lactams, we will also report the % of time above *X* × MIC where % is predefined as 25%, 50%, 75% or 100% and *X* is specified as 1, 2, 3 or 4. Note that 75% > 4 × MIC overlaps with the primary pharmacometric endpoint. These predefined secondary endpoints are part of an exploratory analysis into the relationship between pharmacometric target attainment and clinical endpoints. A post-hoc analysis will be performed to investigate the relationship between clinical endpoints, in particular clinical cure, and pharmacometric target attainment.

### Secondary clinical endpoints

It is important to note that while patients are initially randomized for a single antibiotic, they often receive a combination of antibiotics for which they may receive personalized dosing using AutoKinetics. Therefore, the analysis of clinical endpoints will be based on group allocation (i.e. AutoKinetics versus control), instead of per antibiotic. Mortality was selected as the primary safety endpoint, and analyzed at hospital discharge, day 28 and 6 months. Further secondary clinical endpoints include the attainment of clinical cure (defined as survival and completion of the antibiotic therapy course without addition of or switch to another antibiotic therapy and no start of a new antibiotic therapy within 48 h), length of ICU and hospital stay, delta Sequential Organ Failure Assessment (SOFA) score at 96 h (between first SOFA score and SOFA score on that day depending on the date of randomization (at admission or at a later date)), days free of ventilator support, days free of prone positioning, days free of hemofiltration, days free of other organ days and days free of delirium (as assessed by routinely monitored CAM-ICU and/or DOS scores), all during ICU admission.

Using the EuroQol EQ-5D-5L, the iMTA Medical Consumption Questionnaire (iMTA MCQ) and the iMTA Productivity Cost Questionnaire (iMTA PCQ) tools, the quality of life, calculated in quality-adjusted life years (QALY), and societal costs, based on the questionnaire results, will be assessed at hospital discharge and after 6 months for all included patients and compared between groups [25–28].

These values will be used for an economic evaluation. Incremental cost-effectiveness ratios (ICERs) will be calculated for the ratio of difference in costs between the study groups to the difference in clinical effects (mortality, length of stay and QALY). Furthermore, cost-effectiveness acceptability curves will be used to determine the cost-effectiveness of AutoKinetics in comparison with usual care for a range of different ceiling rations.

Finally, physician satisfaction, as well as their compliance with the decision support software, will be assessed using structured interviews and questionnaires. For quantification of physician satisfaction, a 5-point Likert scale will be used. For quantification of compliance, the log files of AutoKinetics will be used to calculate the percentage of AutoKinetics dose recommendations followed and relate this to potential explanatory factors including the antibiotic group and deviation from standard dosing.

### Calculation of pharmacometric target attainment

Target attainment will be evaluated based on the antibiotic plasma-level measurements at the various sampling time points. The same models that will be used by AutoKinetics to provide dosing advice will be used to generate the full course of antibiotic plasma levels over time, using a Bayesian approach. We will use dedicated pharmacometric software (NONMEM® Version 7.4.3; ICON Development Solutions, MD, USA) to perform these estimations. We will primarily use the total concentration for analysis of pharmacometric endpoints. However, we will explore the effect of using the free fraction concentration of antibiotics for those endpoints by using the available measured free fraction concentrations.

### Principles of statistical analysis

Statistical analysis will be performed using R statistical software. Primarily, an intention-to-treat analysis will be performed. A per-protocol analysis has also been planned, with group allocation determined by the actual dose regimen followed. Descriptive statistics of quantitative data (continuous and categorical) will include the mean, median, standard deviation and interquartile range where appropriate. Missing data will be handled using multiple imputation methods. Mixed-effects logistic models will be used for the analysis of binary endpoints. Mixed-effects linear models will be used for analysis of the log odds transformation of fraction-based outcomes. The between-group comparison for continuous outcomes will be assessed using unpaired *t* tests, the Mann–Whitney *U* test or linear mixed-effect models where appropriate. As already explained, it is theoretically possible that patients are included in two different groups at two different sites. For these patients, only the pharmacometric endpoints will be evaluated.

Time-to-event data will be analyzed by Kaplan–Meier/log-rank analysis and univariate and multivariate Cox proportional hazards regression analysis. The between-group comparison of the EQ-5D-5L scores at hospital discharge and after 6 months will be conducted using a Mann–Whitney *U* test or an unpaired *t* test. Differences between discharge and 6-month utility scores will be analyzed using the Friedman test. Similar analysis techniques will be used for iMTA MCQ and iMTA PCQ measurements.

### Regression model analysis

For the primary endpoint a separate logistic regression model will be used for each antibiotic group, thus aiming for a comparison of *n* = 42 versus n = 42. These models will include the stratification factors (gender, age, center) used in the randomization process and treatment allocation. As already outlined, we opted not to stratify randomization for other factors to avoid treatment delay. Therefore, we intend to also use a regression model to account for potentially modifying effects on other endpoints. A univariable regression analysis will be conducted on all dependent variables and *p* < 0.25 will be used for inclusion in a multivariable model. We predefine these variables as follows:

- Acute Physiology, Age, Chronic Health Evaluation (APACHE) classification, specifically the APACHE IV and or SOFA score as a measure of disease severity
- Prior antibiotic treatment (measured in hours)
- Time since onset of illness (based on documented history)

### Predefined subgroup analysis

If the sample size permits, we will perform subgroup analysis by adding these subgroups as terms in the mixed-effect models used for the whole group analysis. Endpoints for these subgroups will be target attainment in the first 24 h, %-TA, time to TA, length of stay and mortality endpoints. The subgroups are defined as follows: fulfillment of the Sepsis-3 criteria, stratification for severity of sepsis (APACHE IV score or SOFA score), type of sepsis (pulmonary, abdominal, central nervous system, soft tissue, urinary tract, endocarditis, other), type of admission (surgical, medical, neurological), presence of renal dysfunction during treatment as well as the presence of renal replacement therapy. Multiplicity will be accounted for using the Fallback procedure to control the overall probability of a type I error and keep the family-wise error rate (FWER) below 0.05.

A predefined subgroup analysis will be performed to analyze the safety of personalized dosing. The endpoints of interest from a safety perspective are target attainment in the first 24 h, %-TA, clinical cure attainment, new occurrence of AKI, days free of CVVH and ICU mortality. Subgroups are made based on the cumulative dose received by the patient in the first 24 h after randomization in comparison to recommended clinical guidelines: underdosing (< 50% of recommended daily dose), similar dosing (dose within 50% and 200%) and overdosing (> 200%). We choose these categories as they coincide with the lower and upper boundaries of the dose recommendations for which AutoKinetics provides an additional safety warning and may prompt the physician to deviate from the dose recommendation by AutoKinetics. As these safety warnings may influence the physician’s decision-making, we will include physician adherence to the recommended dosing advice as a feature in the model. Furthermore, we include the exposure to each antibiotic as a fixed effect and interaction term in the mixed-effect model.

### Full antibiotic analysis

An additional analysis will be performed for all treatment courses for each antibiotic, including those for which patients were not primarily randomized. An antibiotic treatment course is defined as first antibiotic administration up to the completion of antibiotic therapy course within the ICU without initiation of the same antibiotic within 48 h. For this secondary analysis we will report the primary endpoint as well as the secondary pharmacometric endpoints. Furthermore, for all antibiotic treatment courses, we will perform a subgroup analysis for which we will add the effect of physician adherence to AutoKinetics advice on the %-TA targets as an interaction term.

### Data collection

Blood samples will be collected in standard ethylenediaminetetraacetic acid (EDTA) vials and stored at 4 °C for a maximum of 24 h. Following centrifugation and protein filtration of selected samples for determination of the free fraction, plasma will be stored at − 80 °C. Drug concentrations will be measured using a validated high-performance liquid chromatography (HPLC) column. We will determine both free and total drug concentrations to account for the effect of protein binding. Drug concentration results are stored in the electronic patient record but remain undisclosed for the duration of the trial. From the start of inclusion, relevant data will be collected automatically from each individual patient from the electronic patient record. Additionally, quality of life and societal costs assessment data are collected on paper using the EQ-5D-5L, iMTA MCQ and iMTA PCQ tools.

Data will be entered into an Electronic Data Capture platform. The quality of life and societal costs will be assessed at hospital discharge and after 6 months among all included patients.

### Data management and monitoring

Societal costs and quality of life survey data are collected on paper while all other data are collected directly from the electronic patient record. Patient data are coded. Data will not be directly traceable to individual patients. The decryption key will be kept separately by the primary investigators in Amsterdam UMC, location VUmc. Blood samples will be stored until final analysis and will be disposed of thereafter. All original data, including survey data and laboratory results, will be stored for 15 years following completion of the trial in accordance with national guidelines. Independent quality officers from the Clinical Research Bureau of Amsterdam UMC, location VUmc will monitor the study according to the regulations described under Good Clinical Practice (GCP).

### Safety monitoring

In this population and within this clinical context, events that can be considered serious adverse events (SAEs), suspected unexpected serious adverse reactions (SUSARs) or serious adverse device events (SADEs) within the context of this study population are death and severe new organ failure. We have defined the latter as a new start of renal replacement therapy, a new start of mechanical circulatory support and a new start of prone position ventilation. All SAEs, SUSARs and SADEs will be reported to the appropriate bodies within the time frames prescribed by national law.

The sponsor/investigator has liability insurance which is in accordance with national law. The insurance applies to the damage that becomes apparent during the study or within 4 years after the end of the study.

### Interim analysis and stopping guidelines

The risk qualification for this study is low risk, but this was upgraded to medium risk as vulnerable patients are involved. In accordance with national law, this implies that a formal Data Safety Monitoring Board is not needed. We will, however, conduct an interim analysis as part of our safety strategy, carried out by one of the authors (LFR) under the supervision of our statistician (TLTK). This interim analysis is focused on mortality, and the occurrence of serious adverse events after randomization will be carried out after inclusion of 50% of patients. Those conducting the interim analysis will be unblinded for those outcomes only. As this is the first trial of its kind, no stopping rules for the primary endpoint have been defined.

Analysis will be performed per treatment group and per antibiotic. Between-group comparison of these clinical outcomes will be assessed using the two-group chi-square test or Fisher exact test where applicable. If the between-group difference is large enough to yield *p* < 0.01, we will immediately halt the trial and consult the appropriate authorities. The level of significance should allow for detection of a mortality difference of 25% versus 39% with 1 – β = 0.8.

### Ethical considerations and dissemination policy

The authors declare that they have no competing interests. Both positive and negative study results will be published, in accordance with scientific integrity standards. All study data will be made available guided by the FAIR principles if reasonable and within the context of relevant laws and privacy regulations. Authorship eligibility follows accepted academic standards. We do not intend to use professional writers.

### SPIRIT diagram and Checklist

**Fig. 3:**
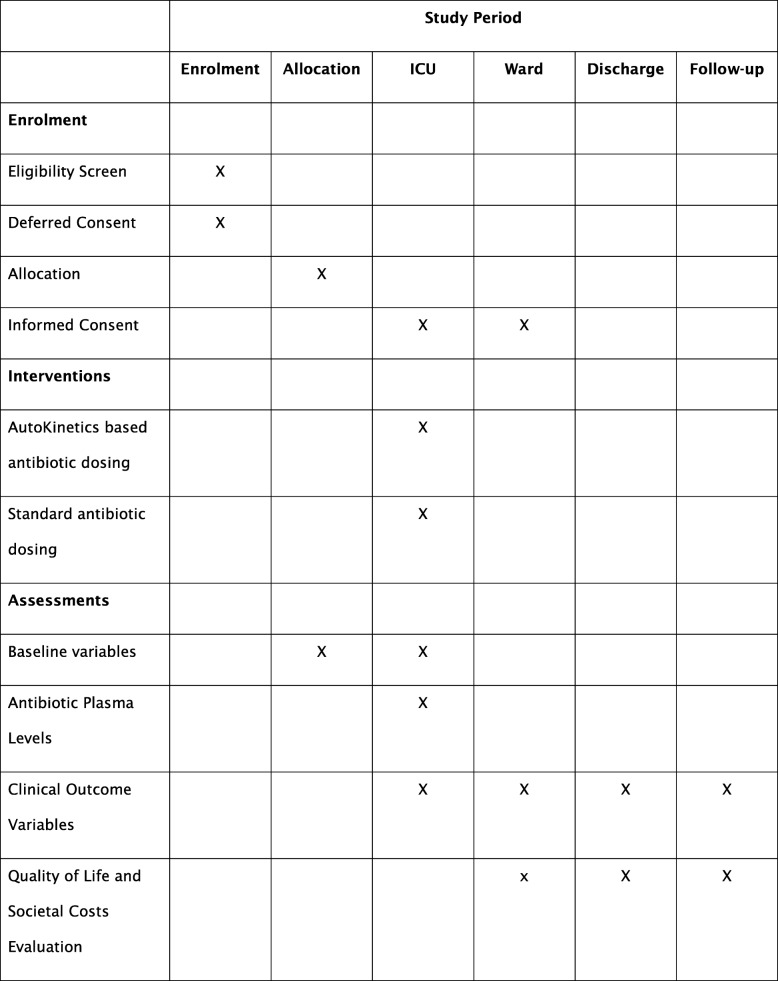
Standard Protocol Items Recommendations for Interventional Trials (SPIRIT) diagram for the trial. ICU intensive care unit

The trial design follows the Standard Protocol Items Recommendations for Interventional Trials (SPIRIT). The SPIRIT Checklist is presented in Additional file 1. The SPIRIT diagram is shown in Fig. 3.

### Trial status

Inclusion started in February 2018. Currently, over 200 patients have been enrolled in the study. The current version of the study protocol is version 5, dated 21 December 2017. We expect recruitment completion before the end of summer 2020.

## Discussion

This is the first study to clinically evaluate bedside data-driven personalized antibiotic dosing in severe sepsis and septic shock. This is important because better antibiotic dosing may lead to better antibiotic plasma-level target attainment, which may improve patient-relevant outcome measures.

Our AutoKinetics software has been designed to optimize antibiotic exposure by reducing the time to adequate antibiotic target and to maintain target attainment for the duration of antibiotic therapy. The live connection with the electronic patient records greatly facilitates real-time and precise dosing decision support as large amounts of data may briskly find their way into the underlying pharmacometric models. This brings therapeutic drug monitoring to the next level, which is that of a stepping stone toward the use of big data and artificial intelligence in the field.

It should be remembered that clinical studies related to dosing decision support and feedback as well as clinical studies assessing the relationship between antibiotic exposure and clinical outcome are scarce. Even though the relationship between antibiotic exposure and bacterial killing is well established in preclinical and clinical studies [10], this paucity of data sharply contrasts the widespread adoption of therapeutic drug monitoring throughout the world. Therefore, the results from our trial may present a significant contribution to the field.

Given the association between early antibiotic administration and survival [2], pharmacometric target attainment on the first day of treatment was chosen as the primary endpoint. Choosing this target is cumbersome as the relatively few preclinical and clinical studies propose significantly varying targets. As a general principle, we aimed to primarily prevent underdosing as this may present the greatest clinical risk to our patients.

It is important to emphasize that this study conceptually involves two targets; that is, the target that the decision support software uses to calculate its dosing recommendations and the target that is used for the evaluation of the pharmacometric endpoints. The importance of this distinction may best be illustrated by considering perfect physician compliance with dosing advice based on a well-validated pharmacometric model. Even under these ideal circumstances, a number of patients would miss their target, although they may not be far off. Therefore, we decided to set the target used for evaluation of the pharmacometric endpoints at 75% of the target used for calculating dose recommendations. As this represents an arbitrary cutoff point and because any cutoff point will remain a subject of debate [29–32], we decided to also explore different cutoff points in our explorative analysis.

An interesting aspect of the trial design is that the study resembles four trials being conducted simultaneously (i.e. one for each study antibiotic). However, patients who are primarily included for a certain antibiotic will also receive dosing advice for the other study antibiotics if prescribed. One advantage of this approach is that while the sample size has been calculated for the primary pharmacometric endpoint, it allows for the grouping of all patients for analysis of the clinical endpoints and thus improves power for those secondary outcomes. A downside of such a design is that it will be more difficult to determine the relative contribution of adequate exposure to individual antibiotics to the clinical endpoints. To address this, we will use mixed regression models to better understand the importance of all relevant contributing and confounding factors.

Originally, the trial design allowed for one more study antibiotic, the β-lactam cefotaxime. However, due to a national shortage prior to the first inclusion and persevering delivery constraints, this antibiotic was not available at the time of writing. As the power analysis was calculated for each antibiotic separately, we chose not to change our intended inclusion rate. We will include patients for this antibiotic should it become structurally available again and if reasonable time remains to implement its model.

In order to avoid any treatment delay, we chose gender and age as stratification factors rather than more sophisticated severity of illness scores, such as APACHE IV or SOFA, as these rely on measurements that are not always immediately available on admission.

For pragmatic reasons, some of the inclusion criteria were based on clinical decisions, such as suspicion of infection and need for vasopressor support. Similarly, only one of either lactatemia or need for a vasopressor was required for enrollment, instead of both. Therefore, strictly speaking, some included patients may be considered severely septic as opposed to in septic shock as defined by the Sepsis-3 criteria [5].

## Supplementary information

**Additional file 1.** SPIRIT 2013 Checklist: Recommended items to address in a clinical trial protocol and related documents.

## Data Availability

The final data set will be deposited in a designated repository under the responsibility of the Department of Intensive Care Medicine at Amsterdam UMC, location VUmc. Please contact the authors to request access.

## Abbreviations

APACHE: Acute Physiology, Age, Chronic Health Evaluation
CAM-ICU: Confusion Assessment Method for the ICU
DALI: Defining Antibiotic Levels in Intensive Care
DOS: Delirium Observation Screening Scale
EDTA: Ethylenediaminetetraacetic acid
EQ-5D-5L: EuroQol five-level, five-dimension instrument
FWER: Family-wise error rate
GCP: Good Clinical Practice
HPLC: High-performance liquid chromatography
ICU: Intensive care unit
iMTA MCQ: iMTA Medical Consumption Questionnaire
iMTA PCQ: iMTA Productivity Cost Questionnaire
MIC: Minimal inhibitory concentration
QALY: Quality-adjusted life years
SADE: Serious adverse device event
SAE: Serious adverse event
SOFA: Sequential Organ Failure Assessment
SPIRIT: Standard Protocol Items Recommendations for Interventional Trials
SUSAR: Suspected unexpected serious adverse reaction
TA: Target attainment
TDM: Therapeutic drug monitoring
